# Evaluation of the Ion AmpliSeq™ PhenoTrivium Panel: MPS-Based Assay for Ancestry and Phenotype Predictions Challenged by Casework Samples

**DOI:** 10.3390/genes11121398

**Published:** 2020-11-25

**Authors:** Marta Diepenbroek, Birgit Bayer, Kristina Schwender, Roberta Schiller, Jessica Lim, Robert Lagacé, Katja Anslinger

**Affiliations:** 1 Department of Forensic Genetics, Institute of Legal Medicine, Ludwig Maximilian University of Munich, Nußbaumstraße 26, 80336 Munich, Bavaria, Germany; birgit.bayer@med.uni-muenchen.de (B.B.); kristina.schwender@med.uni-muenchen.de (K.S.); roberta.schiller@med.uni-muenchen.de (R.S.); katja.anslinger@med.uni-muenchen.de (K.A.); 2 Human Identification Group, Thermo Fisher Scientific, 180 Oyster Point Blvd, South San Francisco, CA 94080, USA; jessica.lim2@thermofisher.com (J.L.); robert.Lagace@thermofisher.com (R.L.)

**Keywords:** forensic phenotyping, HIrisPlex-S, massively parallel sequencing, next-generation sequencing, ancestry, appearance, ancestry prediction, phenotype prediction

## Abstract

As the field of forensic DNA analysis has started to transition from genetics to genomics, new methods to aid in crime scene investigations have arisen. The development of informative single nucleotide polymorphism (SNP) markers has led the forensic community to question if DNA can be a reliable “eye-witness” and whether the data it provides can shed light on unknown perpetrators. We have developed an assay called the Ion AmpliSeq™ PhenoTrivium Panel, which combines three groups of markers: 41 phenotype- and 163 ancestry-informative autosomal SNPs together with 120 lineage-specific Y-SNPs. Here, we report the results of testing the assay’s sensitivity and the predictions obtained for known reference samples. Moreover, we present the outcome of a blind study performed on real casework samples in order to understand the value and reliability of the information that would be provided to police investigators. Furthermore, we evaluated the accuracy of admixture prediction in Converge™ Software. The results show the panel to be a robust and sensitive assay which can be used to analyze casework samples. We conclude that the combination of the obtained predictions of phenotype, biogeographical ancestry, and male lineage can serve as a potential lead in challenging police investigations such as cold cases or cases with no suspect.

## 1. Introduction

Forensic genetics currently stands in front of a new era of DNA analysis as Massively Parallel Sequencing (MPS) is becoming a more commonly used tool for DNA analysis. The enhanced multiplexing capabilities of MPS technology coupled with the ability to analyze a variety of marker types has led to increased research and use of single nucleotide polymorphisms (SNPs) to predict externally visible characteristics (EVCs) and biogeographical ancestry (BGA) from a DNA sample [1,2,3,4,5,6,7,8,9,10,11,12,13,14,15]. To implement the new capabilities in DNA testing, legal changes are obligatory for SNP analysis by MPS to be applied in new cases. Forensic DNA phenotyping (FDP) concerns coding DNA and goes beyond the methods used so far, which are based on testing non-coding regions. The latter provides investigators with the forensic golden standard of an STR profile used to associate a suspect with a crime scene. Forensic phenotyping has arisen as a tool that can be used in situations where there is no suspect. In Germany, a legal change was introduced at the end of 2019 which allows for a forensic specialist to be asked to analyze a casework sample for the eye, hair, and skin color and biological age of the unknown individual who contributed to the trace. A special situation occurs in the federal state of Bavaria where the law exists in an expanded form and it also includes the prediction of one’s ancestry with stipulations that the testing can only be performed in particular cases, such as when serious danger is expected [16]. Due to the ongoing legal changes, the scientific development of forensic DNA phenotyping (FDP) must be followed by the evaluation of the usefulness of those methods when being challenged by actual casework samples. What matters is not only the number and type of markers used or how sensitive and reliable the marker sets are, but also the accuracy of data interpretation and how clearly the results can be presented to law enforcement to minimize bias in investigations. Therefore, we developed the Ion AmpliSeq™ PhenoTrivium Panel, an assay combining ancestry and phenotype-associated SNPs. The panel was tested on known reference samples and real casework samples, as there is a limited number of studies concerning the latter. Our study presents a complex evaluation of one of the most important questions: are the obtained EVC and BGA predictions reliable for forensic investigations?

## 2. Materials and Methods

### 2.1. Panel Design

The Ion AmpliSeq™ PhenoTrivium Panel comprises 320 markers allowing for the prediction of BGA, appearance, and y-chromosomal lineage. BGA and EVC markers were selected from the available literature for a total of 200 autosomal SNPs, from which 163 ancestry SNPs overlap with the Precision ID Ancestry Panel (Thermo Fisher Scientific, Waltham, MA, USA) [5] (rs6464211 and rs12439433 were not used) and 41 phenotype SNPs correspond with the HIrisPlex-S Panel [11] (four markers overlap with the ancestry set). The Y-SNPs chosen cover 20 major haplogroups from the basic Y chromosome phylotree from the International Society of Genetic Genealogy (ISOGG, June 2019) and also include 100 subhaplogroups for better phylogenetic resolution. All 320 markers were submitted to and designed using the Ion AmpliSeq Designer pipeline (www.ampliseq.com). The design was ordered as a single primer pool, containing all BGA, EVC, and y-chromosomal markers, at a 2X primer pool concentration. All 320 SNPs were covered by 196 autosomal targets with a mean amplicon length of 78 bp and 113 Y-chromosomal targets with a mean amplicon length of 217 bp.

### 2.2. Reference Samples

A reference set of samples was collected from volunteers living in in the area of Munich, Germany, following approval given by the Bioethical Commission (reference number 18-870) from the Ludwig Maximilian University of Munich. Buccal swabs from volunteers were taken and they were asked to fill out a questionnaire where they self-declared ancestry using a given family tree (down to the grandparents, with an additional column for those with knowledge about previous ancestors) and self-described physical appearance (pictures of the iris, the back of the head/roots, and the forearm were taken for comparison). All samples were anonymized by assigning numbers to the collected samples immediately following material collection. A total of 140 samples (from 62 males and 78 females) were used for this study. Based on the provided data, 125 individuals were classified as European (84 from Germany), 10 as non-European, and five as admixed.

### 2.3. Sensitivity Study

A buccal swab sample from a male with known phenotype and ancestry was selected for the study. Serial dilutions (1 ng, 500 pg, 250 pg, 125 pg, 62 pg, 31 pg, 7 pg) were prepared and amplified in triplicate.

### 2.4. Casework Samples

Casework samples were used for a blind study to assess the reliability of phenotype and ancestry predictions. Scientists involved in the data analysis and interpretation had no knowledge about the phenotype and ancestral origin of the DNA donors. The results of the blind study served as an evaluation of the interpretation pipeline. Altogether, 17 casework samples were collected: 15 samples (13 blood and 2 bones) from autopsies performed at our Institute (with permission from the Bioethical Commission) and two samples from an actual investigation, submitted for phenotyping by the police. All casework samples were amplified in duplicate. For the 13 blood samples, reference phenotype and ancestry data were based on photos taken during the autopsy and information provided by the police about the place of birth. For the two bone samples, the place of birth was the only available data. No reference phenotype and ancestry data were available for the trace samples submitted by the police as they originated from unknown perpetrators. All collected casework samples were of male origin.

### 2.5. Library Preparation and Sequencing

For all samples used in the study, genomic DNA (gDNA) were extracted on the Maxwell^®^ RSC 48 Instrument using the Maxwell^®^ FSC DNA IQ™ Casework Kit as recommended by the manufacturer (Promega). The extracts were quantified using a Quantifiler™ Trio DNA Quantification Kit (Thermo Fisher Scientific) as recommended by the manufacturer. The results were used to assess possible inhibition, calculate the Degradation Index (DI), and to perform further dilutions of the sample. The samples were diluted to the recommended DNA input (1 ng), while maximum input was used for samples <1 ng DNA.

All sample extracts were subjected to manual library preparation using the Precision ID Library Kit and IonCode™ barcode adapters following the manufacturer’s protocol for Custom Ion AmpliSeq™ SNP Panels (Thermo Fisher Scientific). The numbers of cycles for target amplification were adjusted based on the DNA input amount, with 20 cycles used for samples greater than or equal to 125 pg of genomic DNA (gDNA) and 23 cycles for samples with less than 125 pg of gDNA. An annealing/extension time of 4 min was used for amplification reactions as recommended by the manufacturer. Libraries were quantified using the Ion Library TaqMan Quant Kit (Thermo Fisher Scientific), diluted (if the concentration was lower, a library was not diluted), and pooled equimolarly to 30 pM for template preparation on the Ion Chef using the Ion S5™ Precision ID Chef & Sequencing Kit. A range of 16–24 samples were pooled per 530 chip and sequenced on the Ion S5.

### 2.6. Data Analysis

Primary sequence analysis was performed on TSS 5.10.1 with TMAP alignment of sample reads against the hg19 genome assembly. SNP genotyping and tertiary analysis, in the form of ancestry prediction and Y-haplogrouping, were performed using the HIDGenotyper-2.2 plugin and Converge v2.2 (Thermo Fisher Scientific). Data analysis was separated into two parts: phenotype prediction (which currently cannot be performed within Converge v2.2) and ancestry prediction by the bootstrapping admixture analysis and Y-haplogrouping features of Converge, in instances where Y-SNPs were relevant. Both analyses were performed using the default analysis thresholds: minimum autosomal coverage of 20 reads, minimum Y coverage of 10 reads, major allele frequency at 95% for homozygotes and 65%/35% for heterozygotes. The thresholds were later adjusted as follows: for the SNPs corresponding with the HIrisPlex-S panel, the analytical coverage thresholds were set based on the HIrisPlex-S panel validation for MPS platforms [17] except for rs10756819 and rs1470608. For these two markers, the coverage thresholds were lowered to a minimum of 100 reads when using more than 100 pg DNA input. For samples with less than 100 pg DNA input, the coverage values from the Breslin paper [17] were used. The minimum coverage to call an SNP was set to 100 reads for the remaining autosomal ancestry SNPs and 50 reads for the haploid Y-SNPs. The heterozygote balance threshold was set to 65%/35% for heterozygotes and 90%/10% for homozygotes. For the sensitivity and casework samples, consensus genotypes from replicates were used to generate a single SNP profile for tertiary analysis.

#### Phenotype and Ancestry Predictions

SNP profiles used for phenotype predictions were generated by Converge after running the HIDGenotyper plugin using a hotspot file (SNP names and positions, reference alleles and variants) containing entries for the 41 SNPs within the HIrisPlex-S (HPS) set. The HIrisPlex-S SNP set contains an indel SNP (rs796296176), in the form of an insertion A, that was manually reviewed and called using IGV 2.7 (Integrative Genomics Viewer) [18]. SNP genotypes were exported from Converge in the form of an Excel file reporting all alleles relative to the forward strand. An in-house Excel workbook was used to convert the Converge output into the input file format required by the HIrisPlex-S Webtool (https://hirisplex.erasmusmc.nl/). Predictions were interpreted according to the HPS user manual shared by the authors (HirisPlexS) [11,19,20]. Sequencing results from the known reference samples were used together with the HPS predictions to establish interpretation guidelines for the casework samples tested.

Ancestry prediction was performed using the bootstrapping admixture analysis feature of Converge using the Precision ID Ancestry Panel Ancestry Frequency File v1.1. The frequency file contains genotype frequencies and population data for 146 SNPs of the Precision ID Ancestry panel and covers seven root populations created by hierarchal clustering of 66 populations from ALFRED based on allele frequencies [21,22]. As 163 of 165 of the Precision ID Ancestry SNPs were included in our panel, this corresponds to 145 SNPs (marked in yellow in Table S1) with available genotype frequencies and population data available for bootstrapping admixture analysis. In the bootstrapping admixture analysis feature of Converge, admixture predictions are made based on a maximum likelihood approach used to predict the most likely admixture proportions across seven root populations (herein referred to as the core admixture algorithm): Africa (AFR), East Asia (EA), South Asia (SA), Southwest Asia (SWA), Europe (EU), America (AME), and Oceania (OCE) [21,22,23]. The predictions are bootstrapped across a random subset of sequenced SNPs, specified by the user in %, with each bootstrapping replication ran through the core admixture algorithm N times using a different subset of SNPs for each replication to capture uncertainty in the predictions. The results are displayed as an average of the bootstrapping replications for each population group and a 95% confidence interval reflecting the probable range of variability of the estimated ethnicity percentages [21,22,23]. The predicted ancestry is presented as a percentage of each population with the corresponding likelihood. Sample admixture was estimated using default settings (50% resampling size and 40 replications) and later adjusted to 75% resampling size and 1000 replications after analyzing the reference samples. To contrast the admixture calculations done by Converge, the same genotypes (145 markers maximum) from all the samples were analyzed with SNIPPER [24,25,26]. The analysis was performed using an available reference set corresponding with the Precision ID Ancestry Panel, which included 2099 genotypes from six populations: Africa (AFR), East Asia (EA), South Asia (SA), Europe (EU), America (AME), and Oceania (OCE). Ancestry classification of the studied individuals in SNIPPER was performed using naïve Bayes and presented on PCA (principal component analysis) graphs. Additionally, population likelihoods were calculated using called FrogAncestryCalc, a recently published and open source software that is a stand-alone version of FROG-kb [27,28,29]. Computations for each sample were performed based on genotypes consisting of a maximum of 163 SNPs comprising the Precision ID Ancestry Panel for which the software contains 96 reference populations. The populations with the highest likelihood were taken into consideration for interpreting the ancestral origin of the samples tested.

Y-haplogrouping was performed in Converge using the custom Y haplogroup analysis feature and a custom Y-SNP haplogroup file for 120 Y-SNPs included in the panel. The file was created based on ISOGG (International Society of Genetic Genealogy) Y-Tree version 14.100, accessed on June 2019 (https://isogg.org/). The file contained the SNP name and position, together with its ancestral and derived allele, the haplogroup it defines, and the corresponding parent haplogroup. The included data were used for the Y-haplogrouping, which was based on detecting mutant SNPs. As the final report, the result was presented as the major haplogroup predicted and the most derived (within the panel) subhaplogroup, reported by Converge. All male samples from the study were also analyzed for Y-STRs (Promega PowerPlex23 System) with Y-haplogrouping using Nevgen (https://www.nevgen.org/) in order to assess Y haplogroup concordance between both methods.

## 3. Results

### 3.1. Coverage and Sensitivity

The sensitivity study consisted of a serial dilution of a reference male sample from 1 ng to 7 pg DNA amplified in triplicate for a total of 24 libraries and sequenced on a 530 Chip. Autosomal marker coverage across the 200 autosomal markers included in the panel varied between 967,808 and 1,241,035 total reads for 1 ng replicates and between 104,629 and 287,668 reads for 7 pg replicates. For the 120 Y-chromosomal markers, the values for 1 ng of DNA input oscillated between 236,690 and 271,058 reads and for 7 pg between 30,327 and 80,114 reads. The mean coverage for each marker is presented in the Supplementary Materials Tables S3 and S4. A detailed summary of the performance of the autosomal markers in the case of coverage and allele balance is presented in the Supplementary Materials Table S7.

From 41 autosomal SNPs associated with phenotype, full consensus profiles were obtained down to 125 pg input, where only one marker, rs1470608, did not meet the coverage threshold (Supplementary Materials Figure S1a). Inter-replicate concordance was observed down to 62 pg. Below that DNA input, discrepant alleles were called between the replicates. Discrepant alleles were identified to be drop-in and drop-out alleles. Drop-in alleles passing the coverage thresholds were designated as false allele calls. Starting with 31 pg, false allele calls and allele drop-outs were observed across all replicates and resulted in incorrect genotyping. Accuracy (AUC) loss for all prediction categories was observed starting with 62 pg of input DNA. The observed AUC loss values ranged between 0.008 and 0.033 for eye color, between 0.001 and 0.044/0.001 and 0.027 for hair color/shade, and between 0.001 and 0.046 for skin color. For all samples, eye and skin colors were predicted correctly as blue eyes and pale to intermediate skin. The only incorrect prediction was observed for the consensus profile of 31 pg input DNA due to a homozygote disparity, where an extra allele causes a heterozygote call compared to the expected homozygote call, in IRF4 (rs12203592) for two of three replicates. At 31 pg input DNA, the individual was predicted to have light brown hair when the correct hair color was blonde.

Of the 159 autosomal SNPs associated with ancestry (four SNPs are shared between ancestry and phenotype predictions which were included in the previous section), no drop-outs were observed down to 125 pg of input DNA. At the lowest amount of input DNA of 7 pg, 74% of SNPs (120 markers) exceeded the calling thresholds and were included in the final profile. The number of SNPs used by Converge for admixture predictions was 145 (max. possible) down to 125 pg and 107 markers at 7 pg of input DNA. Discordances between replicates were observed starting at 31 pg and at 7 pg they were observed for 12% of the markers. Incorrect calls passing the genotyping thresholds were observed, starting with 15 pg of input DNA (Supplementary Materials Figure S1b). Admixture predictions from Converge and SNIPPER were correct for all DNA amounts tested and suggested to be of 100% European origin.

From 120 Y-chromosomal SNPs, four markers (P305, M124, M123, and M54) dropped out completely (Supplementary Materials Figure S1c) and two markers, M31 and D-F6251, started to underperform in terms of coverage below 62pg of DNA input. The consensus haplotype at 7 pg of DNA input consisted of 87 Y-SNPs and two markers had an incorrect allele called. The Y haplogroup was predicted as major R and R1b1a1b (R-M269) as the most derived subhaplogroup and as R only down at 7 pg. Y-STR analysis for the same sample using the PowerPlex Y23 System and Nevgen suggested haplogroup R1b1a1b1a1a1 (R-U106).

### 3.2. Reference Samples

#### 3.2.1. Phenotype Predictions

For phenotype predictions, the comparison data consisted of reference photos and a self-described appearance. In the case of hair color, 40 individuals were excluded due to lack of, grey, or dyed hair (the provided data were taken under consideration but not used as final reference due to the subjective color understanding). The outcome of the predictions is presented in Table 1. For the eye color, the highest *p* value was taken as the predicted color. If the highest *p*-value did not exceed 0.5, the prediction was called inconclusive. For hair and skin color, the prediction model presented by the authors was used to group the individuals as presented in Table 1. Overall, 88%, 78%, and 95% of eye, hair, and skin color predictions were correct, respectively, and those values generally correspond with the ones obtained from the validation of the panel by the authors [19,30,31,32].

#### 3.2.2. Ancestry Predictions

##### European Samples

**Figure 1:**
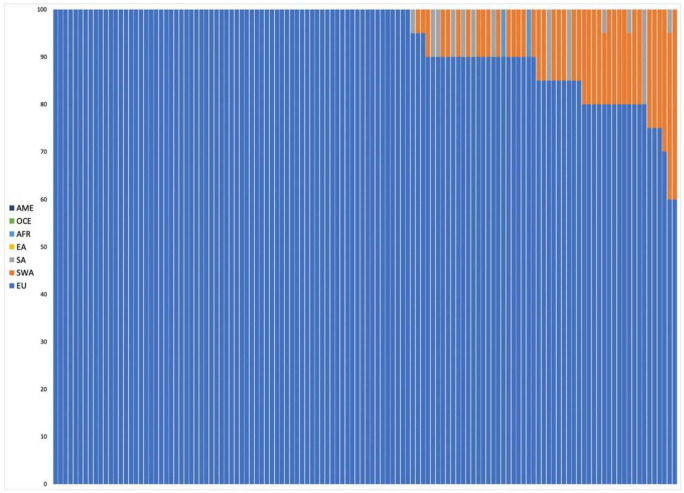
Plot presenting ancestry predictions for the European individuals calculated by Converge Software using the bootstrapping admixture analysis (20–22). The predictions were bootstrapped across a random subset of sequenced single nucleotide polymorphisms (SNPs) multiple times, with each bootstrap sampling ran through the core admixture algorithm, producing an average prediction (summing up to 100%) result from all replications, presented here as a single bar, corresponding with a single individual. Samples were sorted by ascending percentage of admixtures detected.

**Figure 2:**
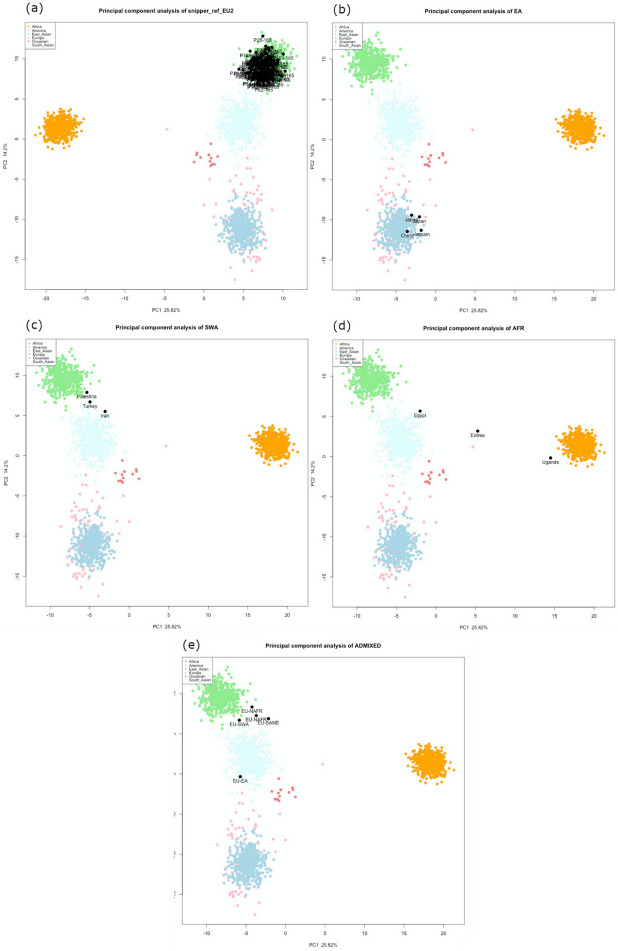
Principal component analysis plots by SNIPPER for all the reference samples. The results are shown separately for individuals classified by the provided data as from (**a**) Europe, (**b**) East Asia, (**c**) Southwest Asia, (**d**) Africa, and (**e**) admixed. The samples are named by their stated origins.

Altogether, 125 individuals with self-declared European ancestry down to the 3rd generation were analyzed. The results of the admixture analysis performed by Converge are presented in Figure 1. All individuals were assigned to EU with some of them showing SWA admixture up to more than 30%. These samples included all the southeastern European individuals and a few German individuals. SNIPPER classified all samples more than a billion times more likely to come from Europe than any other population included in the reference set (Figure 2a).

**Figure 3:**
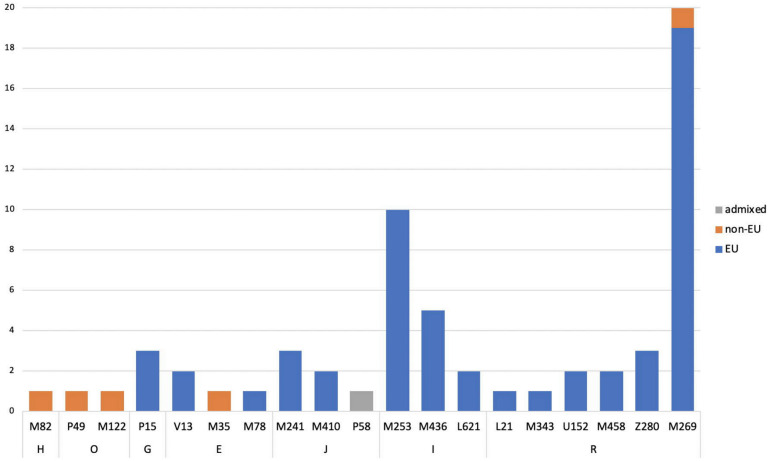
Summary of the Y-lineage analyses on the 62 male individuals of the reference study. For all samples, Nevgen’s Y-STR haplotype-based haplogroup predictions were verified by sequencing of Y-SNP markers. In 22% of the cases, fully concordant results were obtained. Furthermore, sequencing placed 64% of the individuals slightly higher in the phylogeny, as the terminal Y-SNP marker suggested by the software was not part of the sequenced marker panel. Finally, 14% of the Y-haplogroup predictions were overruled by sequencing (SNPs suggested by Nevgen were sequenced and had ancestral state). While the major haplogroup assignments proved stable, the subhaplogroup assignments changed in these cases.

As the European genetic landscape is very complex, FROG analysis confirmed the previous estimates without providing a better differentiation. However, it was observed that for the individuals with European ancestry of at least 90%, the highest likelihoods were represented by major EU populations (e.g., Irish, Danes, Hungarians). For the individuals with inferred SWA admixture, the populations suggested by FROG included, among different EU populations, Turks or ethnic groups like Ashkenazi Jews (only two of them had confirmed Jewish ancestry). The Y-chromosome SNPs established for 56 European males are described as common in EU (Figure 3). In only some cases, the subhaplogroups represented the lineages known to be more frequent among particular populations and corresponded with the described heritage, like I-L621 (Romania), R-L21 (England), or R-M458 (Czech Republic) [33,34,35].

##### Non-European Samples

The summarized results of ancestry prediction for ten non-European samples are presented in Table 2 (admixtures by Converge, population likelihood ratios by SNIPPER, population likelihoods by FROG, Y-lineage) and Figure 2b–d (PCA by SNIPPER). Both Converge and SNIPPER correctly predicted four samples as EA. Analysis by Converge’s bootstrapping admixture algorithm for three SWA samples showed admixture between SWA and other populations. One sample with self-reported ancestry from Palestine showed admixture with EU, one sample (from Iran) showed admixture with SA, and one sample (from Turkey) showed admixture with both EU and SA. For these samples, SNIPPER did not detect the same admixtures and they were all assigned to one population only—EU or SA (Figure 2c)—however, the LR values for two samples, namely Turkey and Iran, were low (Table 2). From three African samples, only one (from Uganda) was predicted as AFR by both Converge and SNIPPER. The East African sample (from Eritrea) showed strong admixture with SWA when analyzed by Converge and was assigned to SA by SNIPPER. The North African individual (from Egypt) was assigned to SWA only by Converge and SNIPPER detected admixture of EU and SA (Table 2).

As provided in the guidelines for FROG-kb, the calculated probabilities do not consider multiple ancestries. Therefore, the results presented here for non-European samples did not always correspond with the detected admixtures but overall, the highest population likelihoods agreed with self-declared ancestry, e.g., “Mainland Japanese” and “Okinawa Japanese” for Japan or “Ethiopian Jews” and “Somalis” for East Africa. The established Y-lineages correlated closely with ancestry predictions based on autosomal markers analysis: e.g., H-M82 for Iran or O-P49 for Japan [36,37].

##### Admixed Samples

The results of the predictions for five samples known to be admixed are shown in Table 3 (Converge, SNIPPER, FROG, Y-lineage) and Figure 2e (SNIPPER). The data provided by the volunteers were used to create “expected” admixtures by referring to seven reference populations. For Sample 1, an individual with European–East Asian (Germany–China) descent, Converge detected very accurate admixture of EU and EA. The same sample was assigned to SA by SNIPPER (Figure 2d, Table 3). Samples 2 and 3 had North African (Tunisia and Algeria) ancestry of 50% and 25% and both were detected by Converge as an admixture of AFR and SWA. Sample 5, with 25% of SWA ancestry (Iran), showed SWA and SA admixture, which corresponds with previously presented results obtained for an individual with Iranian origin. Sample 4 was the only sample with an unexpected result as the estimated 50% of South American (Guyana) heritage was predicted by Converge to be of AFR descent only. For samples 3–5, no admixture was detected by SNIPPER, but for three of them, the calculated LR values were low (Table 3).

The population likelihoods calculated by FROG-kb did not adequately reflect the calculated admixtures and for one sample, the results did not correspond with expected reference populations. For an individual of European–East Asian ancestry, the highest likelihoods were represented by rare ethnic groups which did not comply with the self-declared ancestry (Germany and Japan). Only one of the admixed samples was male and the analysis of Y-lineage revealed a haplogroup found rarely in Europe, namely J-P58 [38,39]. The paternal lineage of this individual was described as Algerian.

### 3.3. Casework Samples

The lowest total coverage across all the markers was observed for the bones (Sample C1 with 485,514 and Sample C2 with 35,928 total reads) and for one autopsy sample with a high degradation index (Sample C11 with 449,210 total reads). The mean coverage for each marker is presented in the Supplementary Materials Tables S5 and S6. The number of markers used and prediction results based on the consensus profiles are summarized in Table 4. All predictions were made based on the reported SNP genotypes and the interpretation pipeline established by the sensitivity and reference sample studies mentioned previously. The combination of the ancestry and phenotype predictions for casework samples were described as they would be compared with reference data if available (Table 5).

Phenotype predictions were possible for all casework samples tested, with the exception of one sample, a bone with 31 pg of input DNA and a consensus profile containing only 12 markers which was not enough for HPS tool to perform a prediction. Accuracy (AUC) loss was observed for skin color prediction for eight samples; however, AUC loss was low (max. 0.003) and did not affect the final predictions. Out of 13 blood samples, four samples had predictions of all phenotypic traits (eye, hair, and skin color) that aligned with the available reference data. For six blood samples, reference data on hair and skin color were only available and the predicted results were in agreement with the reference data. The rest of the samples had no comparison data (decay, skeletonization, crime scene).

The final ancestry prediction was based on the results of admixture analysis (Converge), population likelihoods calculation (FROG), and Y-lineage analysis (Converge). The predicted phenotype from the HIrisPlex-S tool was also taken into consideration. Ancestry assignment was described on two levels, inter- and intracontinental (Europe, Africa, Asia, America, Oceania) or admixed, and by adding the relative probability of the prediction as “high” or “likely” depending on the obtained data. Predictions were designated as “high” if all ancestry and the phenotype estimates were in agreement. For predictions classified as “likely”, the phenotype prediction strongly correlated with only part of the ancestry data (for example, admixed individuals). Of 17 casework samples, one was not interpreted (Sample C2), a bone sample for which only 40% of the ancestry markers were typed. From the remaining 16 samples, nine were assigned biogeographical ancestry on an inter/intracontinental level and seven were described as admixed. The comparison of 15 autopsy samples to the available ancestry reference data revealed that for 12 samples, the predicted origin of the individuals corresponded with the place of birth and one sample had an incorrect prediction. For the sample with an incorrect prediction, the genetic data suggested European ancestry, however the self-reported place of birth was Brazil (no further information about the individual was available).

## 4. Discussion

The first studies introducing phenotype and ancestry prediction to forensics [3,20,23,40,41,42,43,44] have prompted the discovery of new markers and methods that have been published in the scientific literature [10,11,12,13,15,21,45]. The continual development of DNA analysis technology goes hand in hand with a discussion beyond DNA itself, leading to a debate about the ethics and laws behind forensic phenotyping [46,47,48,49,50,51]. Despite great interest in the topic, concerns have been raised against the new forensic approach. The main purpose of predicting phenotype and ancestry is to include DNA as an additional, or sometimes the only, “eyewitness” and to compare the data it provides to the information gathered by police. The understandable concern is that predictions might be incorrect or wrongly interpreted and they could negatively affect the investigation by introducing bias. Predictions regarding the biogeographical ancestral origin of a sample are very complex, since the “ancestry” of an individual can be interpreted on many different levels. The results of ancestry prediction by DNA analysis alone only provides information about a person’s biogeographical history at the genetic level. Non-genetic events such as a change in the place of residence, a change in citizenship, or an adoption (also in previous generations) are not always common knowledge. Additionally, in the case of phenotype predictions, it must be taken into account that the results and their interpretation can be faced with some discrepancies due to biased perceptions. As an example, hair color is subjective and predictions of dark blonde hair color can be described subjectively as brown. Hair and eye color can also be artificially altered by dying one’s hair or wearing colored contacts. However, if we consider skepticism towards the information provided by DNA, we should also consider the limitations of relying on real eyewitnesses only [52,53]. With no solution being absolutely faultless, the final question is if DNA can lead or mislead the search for a suspect when dealing with cold cases. In an attempt to investigate this question, we present not only the results of testing our custom SNP panel on known reference samples but also a blind study performed on real casework samples. This approach was used in order to better understand the value of ancestry and phenotype predictions, as well as evaluate the accuracy of the information that would be provided to police investigators.

As mentioned previously, the accuracy of the predictions depends on many factors including the number and type of markers used and how sensitive and reliable those sets are. Until recently, markers associated with phenotype and ancestry were studied separately, with the exception of the commercially available ForenSeq DNA Signature Prep Kit (Verogen). The newly published panel from the VISAGE consortium [53,54] is the first MPS-based solution combining phenotype and ancestry predictions, compatible with two MPS platforms (Ion S5, Thermo Fisher Scientific and MiSeq FGx, Verogen). The assay consists of 153 autosomal phenotype and ancestry-informative SNPs, compared to 200 autosomal and 120 Y-chromosomal targets included in the presented Ion AmpliSeq Phenotrivium panel. The sensitivity studies for the VISAGE assays showed that no drop-outs were observed down to 100pg for the AmpliSeq assay and down to 125 pg for the MiSeq platform [54,55]. In the presented study, only one autosomal SNP (rs1470608) dropped out at 125 pg input due to low coverage. Observations about the weak amplification rate of rs1470608 have been previously reported in the development of the SNapShot and MPS versions of the HIrisPlex-S (HPS) Panel [11,17,56]. However, the drop-out of rs1470608 causes minimal AUC loss of 0.001 and does not affect the final skin color prediction. We observed that starting with 31 pg of DNA input, drop-in alleles passed the thresholds to call an SNP, causing incorrect genotyping. When working with the same DNA quantity obtained from a degraded bone, a consensus profile did not allow for phenotype prediction, demonstrating the strong impact of DNA quality. A study from Kukla-Bartoszek and Szargut [56] also presents the results of forensic phenotyping of high degraded bone samples and suggests that full genotypes can be obtained down to 50 pg of DNA input. In the case of the mentioned study, an additional challenge was a lack of reference data for most of the individuals (almost all of the remains belonged to the victims of communism crimes in Poland in the 1950s) so the reliability of the predictions could not be entirely evaluated.

Based on our results and HPS interpretation guidelines, we were able to establish an internal pipeline to be used for unknown samples. The prediction model developed by HPS authors has undergone forensic developmental validation and shows an accuracy of 80% for eye color, 77 % for hair color, and 80% for skin color prediction. The values obtained through our own internal validation were similar or higher than the suggested values obtained in the HPS developmental validation. The predictions we obtained for the casework samples used in this study were compared with the available premortem data about the studied individuals and suggested a high degree of correctness of predicted phenotypes.

In addition to concerns associated with the use of forensic DNA phenotyping, predicting one’s biogeographical ancestry for criminal investigations has additional reservations due to the possibility of investigational bias. As previously mentioned, the concept of “ancestry” is complex and can lead to many misunderstandings, which has been well recognized by scientists [57,58]. Naturally, it raises more concerns when considered as a potential investigative lead in police work. In the case of forensics, the complexity of one’s ancestry suffers from an additional factor, which is the quality of DNA that forensic scientists deal with. The detection of ancestry admixture and the understanding of predicted outcomes can be affected by incorrect genotyping caused by SNP drop-out, and allele drop-in and drop-out, commonly encountered with degraded and/or low-input DNA. However, over the years, a few compact sets of SNPs were developed and suggested for forensic purposes [3,44,59,60], accompanying different analysis approaches that are recommended for biogeographical ancestry prediction [61,62,63,64]. In the Ion AmpliSeq™ PhenoTrivium panel, 163 autosomal ancestry-informative SNPs from the Precision ID Ancestry Panel were included, which has been tested on various ethnic groups [65,66,67,68]. Among the markers within the panel, 55 SNPs are known as the KiddLab Set, which are also present in the ForenSeq DNA Signature Prep Kit [69,70] and the VISAGE assay [54,55]. The remaining markers correspond with a set established by the Seldin group [59,71]. A widely known golden standard in population structure analysis and ancestry inference is an open source software known as STRUCTURE by the Pritchard Lab, Stanford University. However, becoming familiar with the software’s algorithm can be challenging for less experienced researchers, especially if they are based solely in forensics and not familiar with advanced population genetics. As also observed by others, the results produced by STRUCTURE can be overinterpreted and this is one of the fears in using ancestry predictions in police investigations [72]. In the presented study, we evaluated the effectiveness and reliability of ancestry predictions based on admixture analysis performed by user-friendly Converge software when using the previously described SNP set. The predictions are based on a maximum likelihood approach that is used to calculate the most likely admixture proportions across the seven root populations of Africa, East Asia, South Asia, Southwest Asia, Europe, America, and Oceania. The predictions are bootstrapped across a random subset of SNPs to capture uncertainty in the predictions. For the validation of the discussed workflow, we collected 140 known reference samples that came from volunteers living in the federal state of Bavaria. Based on the information provided by volunteers, we divided the collected samples in to three categories: Europe (EU), non-European (non-EU), and admixed. All of the individuals assigned to the first group were correctly predicted to be European, with some of them showing Southwest Asian admixture (up to over 30%). The high SWA admixtures were inferred for around 20% of the samples declared to be German (all from South Germany) and among Southeast Europeans (Albania, Bulgaria, former Yugoslavia). The Bayesian and PCA analysis done by SNIPPER assigned all the samples as 100% EU but the available reference grid used for the predictions does not include SWA populations. The detection of Southwest Asian admixture in European samples, especially in the southeast region of Europe, corresponds with similar findings from other studies and may be explained as a consequence of earlier human migrations when the farmers from Anatolia and Western Asia spread throughout Europe [73,74,75]. Of the individuals classified as non-European, none declared admixed ancestry. For almost all of the samples, Converge detected admixture of two or even three reference populations; however, the results reflected the genetic origin of the samples when historical migration patterns are taken into account. The admixtures detected for the studied individuals correspond with extensive studies concerning the populations of interest [76,77,78,79,80,81]. Additionally, for the remaining European individuals with confirmed mixed ancestry, the analysis showed that the detected admixtures reflect their non-European origin. Only one sample showed a surprising prediction, namely the individual of European and South American ancestry. The obtained results can be explained not by the data provided in the questionnaire but by population studies that try to explain the complexity of ancestry by understanding migration patterns and historical events. For this sample, an admixture of European and African ancestry was predicted by Converge, in contrast to the expected admixture of European and American reported by the individual. Volunteers were asked to specify any additional details about their heritage but were not expected to be familiar with their complete genetic heritage. The presence of African lineages in Latin America is a well-studied topic [82,83] and corresponds with the African admixture detected for the studied individual.

The complexity of admixture detection and interpretation is a complicated issue from a scientific point of view and can be more problematic when the information may be shared with police investigators for use in criminal investigations. Therefore, based on the studies performed, we included ancestry inference in the form of relative population likelihoods calculated by FROG-kb and, for male individuals, paternal lineage analysis results in our final interpretation pipeline, all in order to have a better understanding of the predicted ancestry due to the complexity of biogeographical ancestry prediction. This approach, combined with phenotype predictions, was tested on real casework samples. The challenging aspects of this study were not limited to the quantity and quality of the DNA, but also the blinded aspect of the study. The comparison of the estimated phenotype and ancestry predictions with available reference data revealed high correctness of the predictions, but also pointed out the possible limitations in using phenotype and ancestry predictions as investigative leads for police.

## 5. Conclusions

This study presents the evaluation of the Ion AmpliSeq™ PhenoTrivium Panel and Converge™ Software for use in forensic investigations. The assay contains 200 autosomal and 120 Y-chromosomal SNPs, allowing for predictions of phenotype, biogeographical ancestry, and male lineage. The panel demonstrated to be a sensitive assay, which provides reliable predictions down to 125 pg of DNA input. Biogeographical ancestry and phenotype predictions were possible down to 62 pg but are to be interpreted with caution. Samples with less DNA, especially degraded ones, were treated as not suitable for forensic phenotyping. The results provide a basis for an analysis pipeline to combine ancestry and phenotype predictions using a combination of Converge™ Software, SNIPPER and Frog-kb for ancestry analysis, and the HIrisPlex-S webtool for phenotype analysis. Y-chromosomal lineage markers added informative data about male individuals and aided in a better understanding of the ancestry predictions. Future research could explore the use of additional haploid markers, such as mitochondrial DNA, together with autosomal markers to assess the amount of informativeness when combining autosomal and haploid markers together for analysis. The Ion AmpliSeq™ PhenoTrivium Panel, covering 200 autosomal markers and 120 Y-SNPs, will be available as a community panel via https://ampliseq.com/.

## Figures and Tables

**Table 1:** Summary of the phenotype predictions for the reference samples. For each phenotypic trait, the mean *p*-values calculated for each HIrisPlex-S category were used to group the predictions as presented. The table also includes a quantitative summary of the predictions.

Mean *p*-Values for Each HIrisPlex-S Category among Tested Reference Samples							Example	Prediction	Number of Predictions per Category (Incorrect Ones in Red)
**EYE COLOR**	**Blue**		**Intermediate**		**Brown**				
	0.900		0.061		0.039		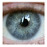	Blue	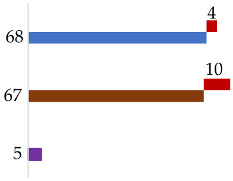
	0.097		0.123		0.780		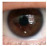	Brown	
	0.336		0.240		0.424		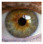	Inconclusive	
**HAIR COLOR**	**Color**				**Shade**				
	**Blond**	**Brown**	**Red**	**Black**	**Light**	**Dark**			
	0.212	0.103	0.678	0.007	0.969	0.031	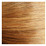	Red	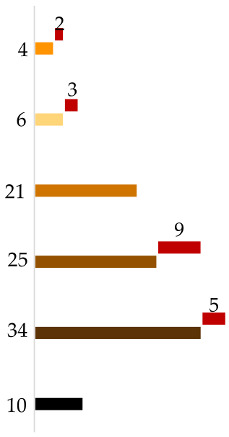
	0.758	0.185	0.041	0.017	0.986	0.014	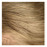	Light blond to blond	
	0.582	0.315	0.066	0.037	0.935	0.065	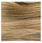	Blond to dark blond	
	0.302	0.553	0.058	0.088	0.821	0.179	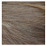	Light brown to brown	
	0.206	0.619	0.012	0.163	0.525	0.475	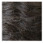	Brown to dark brown	
	0.013	0.330	0.000	0.657	0.026	0.974	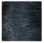	Dark brown to black	
**SKIN COLOR**		**Very Pale**	**Pale**	**Inter.**	**Dark**	**Dark/** **Black**			
		0.204	0.705	0.091	0.004	0.000	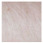	Very pale to pale	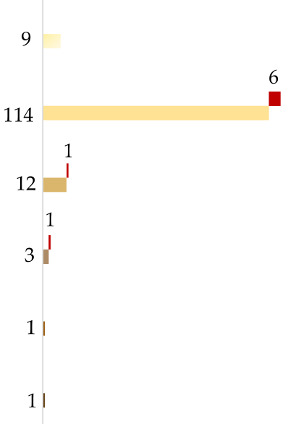
		0.046	0.492	0.454	0.008	0.000	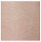	Pale to intermediate	
		0.005	0.068	0.878	0.050	0.004	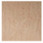	Intermediate	
		0.003	0.021	0.497	0.458	0.024	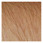	Intermediate to dark	
		0.001	0.006	0.262	0.721	0.010	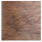	Dark	
		0.000	0.000	0.000	0.001	0.999	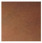	Dark to black	

**Table 2:** Summary of the ancestry prediction for non-European samples including admixture analysis by Converge, likelihood ratio (LR) calculated by SNIPPER using Naïve Bayes, population likelihoods by FROG-kb and Y-lineage analysis (most derived subhaplogroup shown; N/A corresponds with female samples).

Admixture (by Converge)	LR (by Snipper)	Population Likelihoods FROG (Highest)	Y-Lineage
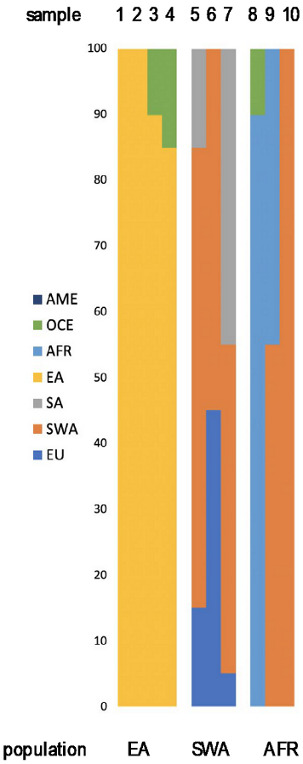	Sample 1 (**Japan**) billion times more likely EA than SA and AME	Sample 1 (**Japan**) Japanese 1.3 × 10^−51^ Mainland Japanese 1.9 × 10^−52^	O1b2 P49
	Sample 2 (**China**) billion times more likely EA than AME and SA	Sample 2 (**China**) Yi (Sichuan) 1.1 × 10^−51^	O2 M122
	Sample 3 (**Vietnam**) billion times more likely EA than AME and SA	Sample 3 (**Vietnam**) Hakka 1.1 × 10^−46^ Lao Long 8.3 × 10^−47^ Mainland Japanese 7.8 × 10^−47^	N/A
	Sample 4 (**Japan**) billion times more likely EA than SA and AME	Sample 4 (**Japan**) Mainland Japanese 7.2 × 10^−53^ Okinawa Japanese 4.4 × 10^−53^ Japanese 2.2 × 10^−53^	N/A
	Sample 5 (**Turkey**) 18.94 times more likely EU than SA and billion than OCE	Sample 5 (**Turkey**) Iranians 2.0 × 10^−41^ Pathan 6.9 × 10^−42^ Turks 3.1 × 10^−42^	R1b1a1b M269
	Sample 6 (**Palestina**) billion times more likely EU than SA and EA	Sample 6 (**Palestina**) Turkish Cypriots 6.1 × 10^−49^	N/A
	Sample 7 (**Iran**) 458 times more likely SA than EU and billion than OCE	Sample 7 (**Iran**) Iranians 3.9 × 10^−48^ Turks 7.2 × 10^−49^	H1a1a M82
	Sample 8 (**Uganda**) billion times more likely AFR than SA and OCE	Sample 8 (**Uganda**) Lisongo 1.2 × 10^−38^ Hausa 1.1 × 10^−39^	N/A
	Sample 9 (**Eritrea**) billion times more likely SA than EU and AFR	Sample 9 (**Eritrea**) Ethiopian Jews 9.1 × 10^−51^ Somalis 1.6 × 10^−52^	E1b1b1 M35
	Sample 10 (**Egypt**) 1.36 times more likely EU than SA and billion more than AME	Sample 10 (**Egypt**) Palestinian Arabs 1.7 × 10^−51^	N/A

**Table 3:** Summary of the ancestry prediction for admixed samples including a graphical presentation of the expected admixture (based on the data provided and referring to reference populations in Converge), admixture analysis by Converge, likelihood ratio (LR) calculated by SNIPPER using Naïve Bayes, population likelihoods by FROG-kb and Y-lineage analysis (most derived subhaplogroup shown; N/A corresponds with female samples).

Expected Admixture (Based on Provided Data)	Predicted Admixture (Calculated by Converge)	LR (by SNIPPER)	Population Likelihoods FROG (Highest)	Y Lineage
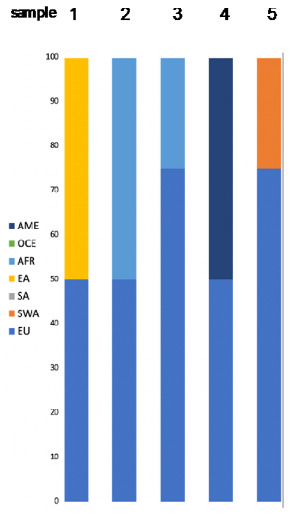	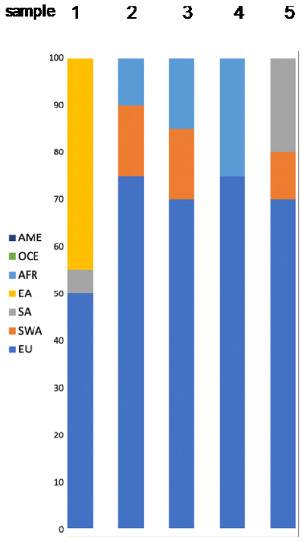	billion times more likely SA than EU and EA	Sample 1 Chuvash 6.2 × 10^–53^ Qinghai Tibetans 1.1 × 10^–53^ Khazaks 7.8 × 10^–54^	N/A
		billion times more likely EU than SA and AME	Sample 2 Italians 7.0 × 10^–48^ Turks 5.7 × 10^–48^ Turkish Cypriots 1.2 × 10^–48^	N/A
		128,027 times more likely EU than SA and billion more than AME	Sample 3 Kairoun,Tunisia 5.9 × 10^–49^ Smar,South Tunisia 5.9 × 10^–50^	J1a P58
		795 times more likely EU than SA and billion more than OCE	Sample 4 Sousse, Tunisia 1.1 × 10^–53^ Kairoun,Tunisia 1.8 × 10^–54^ Smar, South Tunisia 1.7 × 10^–54^	N/A
		221,461 times more likely EU than SA and billion more than AME	Sample 5 Mixed EU 4.8 × 10^–46^ Russians 2.7 × 10^–46^ Finns 1.6 × 10^–46^	N/A

**Table 4:** Summary of panel performance on challenging casework samples, together with detailed values obtained for phenotype, ancestry, and Y-lineage analysis. For phenotype, the highest *p*-values are bolded. For admixtures, the percentage of each reference population detected is presented. For population likelihoods, the highest values marked by FROG are presented. For Y-lineage, major haplogroup and subhaplogroup reported by Converge are presented. DI = Degradation Index.

Sample and Material	DNA Input (DI)	Used SNPs Maximum:			*p*-Values				Admixture Converge (% Mean)	Population Likelihoods FROG (Highest)	Y-Lineage
					Eye Color	Hair Color	Hair Shade	Skin Color			
		163 Ancestry	41 Phenotype	120 Y-SNPs	Blue Inter Brown	Blond Brown Red Black	Light Dark	V Pale Pale Inter Dark B-Dark			
C1 bone	125 pg (1.4)	163	30	110	0.001 0.017 0.982	0.097 0.645 0.001 0.257	0.052 0.948	0.000 0.000 0.001 0.192 0.807	51.50 SWA 48.50 AFR	Ethiopian Jews 5.7 × 10^−52^	Major: E Subhap: E1b1b1 (M35)
C2 bone	31 pg (1.2)	66	12	29	NA				NA	NA	NA
C3 trace	62 pg (1.6)	154	40	107	The exact *p*-values cannot be published due to an ongoing investigation						
C4 trace	125 pg (1)	163	40	113							
C5 blood	1 ng (1.1)	163	41	116	0.932 0.046 0.021	0.433 0.046 0.519 0.002	1.000 0.000	0.098 0.654 0.249 0.000 0.000	100 EU	Danes 4.5 × 10^−45^ Mixed EU 4.0 × 10^−45^ Irish 3.8 × 10^−45^ Hungarians 3.7 × 10^−45^	Major: R Subhap: R1a1a1b1a2 (Z280)
C6 blood	1 ng (0.9)	162	41	116	0.000 0.002 0.998	0.002 0.301 0.000 0.697	0.002 0.998	0.000 0.000 0.000 0.003 0.997	100 AFR	Yoruba 3.1 × 10^−34^ Zaramo 4.7 × 10^−35^ Lisongo 3.5 × 10^−35^	Major: E Subhap: E1b1a1 (M2)
C7 blood	1ng 1	162	41	116	0.000 0.002 0.998	0.003 0.264 0.000 0.733	0.007 0.993	0.000 0.000 0.000 0.060 0.940	60.64 SWA 39.36 AFR	Ethiopian Jews 4.1 × 10^−57^	Major: E Subhaplo: E1b1b1 (M35)
C8 blood	1 ng 0.9	161	41	116	0.000 0.002 0.998	0.003 0.425 0.000 0.571	0.003 0.997	0.000 0.000 0.057 0.923 0.020	55.18 AFR 41.82 SWA	Somalis 6.7 × 10^−57^ Ethiopian Jews 6.6 × 10^−57^	Major: T Subhaplo: T1a (M70)
C9 blood	1 ng 1.6	163	41	115	0.000 0.007 0.993	0.007 0.246 0.000 0.747	0.014 0.986	0.000 0.000 0.998 0.002 0.000	92.40 EA 7.60 EU	Hakka 3.9 × 10^−54^ Taiwanese Han 1.0 × 10^−54^ SF Chinese 5.3 × 10^−55^	Major: R Subhaplo: R1b1a1b (M269)
C10 blood	1 ng 2.2	161	41	115	0.000 0.003 0.997	0.001 0.133 0.000 0.866	0.003 0.997	0.084 0.000 0.976 0.024 0.000	95.06 EA 4.94 SA	Lao Long 4.2 × 10^−53^	Major: O Subhaplo: O1b1 (F2320)
C11 blood	1 ng 7	162	41	108	0.911 0.057 0.032	0.576 0.379 0.003 0.042	0.917 0.083	0.021 0.489 0.475 0.011 0.004	92.84 EU 5.26 SWA	Irish 2.7 × 10^−47^ Danes 1.4 × 10^−47^ Russians 1.1 × 10^−47^	Major: R Subhaplo: R1b1a1b (M269)
C12 blood	1 ng 1	163	40	116	0.00 0.004 0.996	0.004 0.311 0.000 0.685	0.004 0.996	0.000 0.000 0.019 0.965 0.016	67.56 SWA 29.35 EU 3.09 SA	Iranians 2.4 × 10^−42^ Palestinian Arabs 2.1 × 10^−42^	Major: I Subhaplo: I2 (M438)
C13 blood	1 ng 1	161	40	116	0.000 0.002 0.998	0.002 0.301 0.000 0.697	0.002 0.998	0.000 0.054 0.000 0.005 0.995	100 AFR	Yoruba 1.1 × 10^−29^ Ibo 4.9 × 10^−30^ Lisongo 2.1 × 10^−0^	Major: E Subhaplo: E1b1a1 (M2)
C14 blood	1 ng 1.3	160	40	115	0.012 0.050 0.938	0.072 0.706 0.001 0.221	0.137 0.863	0.000 0.000 0.210 0.339 0.452	56.77 EU 27.40 SA 15.83 OCE	Iranians 9.1 × 10^−53^	Major: R Subhaplo: R1a1a1b2 (Z93)
C15 blood	1 ng 1	163	41	116	0.000 0.003 0.997	0.002 0.211 0.000 0.787	0.004 0.996	0.007 0.020 0.644 0.332 0.008	76.32 AME 15.06 SWA 8.62 AFR	Ecuadorian Mestizo 2.8 × 10^−69^	Major: Q Subhaplo: Q1b1a1a (M3)
C16 blood	1 ng 0.8	163	40	116	0.028 0.073 0.899	0.087 0.492 0.001 0.420	0.169 0.831	0.113 0.268 0.550 0.045 0.024	51.54 SWA 44.23 EU 4.23 SA	Druze 7.9 × 10^−48^	Major: J Subhaplo: J2a (M410)
C17 blood	1 ng 0.8	163	40	116	0.000 0.003 0.997	0.001 0.087 0.000 0.912	0.003 0.997	0.000 0.000 0.997 0.003 0.000	95.85 EA 4.15 OCE	Koreans 5.5 × 10^−54^ Japanese 3.0 × 10^−54^	Major: D Subhaplo: D1b (M55)

**Table 5:** Summary of final predictions compared to available reference data.

Sample	Phenotype Prediction	Phenotype (Photo)	Ancestry Prediction	Place of Birth
C1	Brown eyes Dark brown to black hair Black skin	No data (body skeletonized)	ADMIXED (AFR-SWA) Likely: East Africa	Eritrea
C2	No prediction	No data (body skeletonized)	No prediction	Eritrea
C3	Brown eyes Light brown to brown hair Pale to intermediate skin	No data (police investigation)	High: Europe	No data
C4	Brown eyes Light brown to brown hair Pale to intermediate skin	No data (police investigation)	High: Europe	No data
C5	Blue eyes Red hair Pale skin	No data (body decayed)	High: Europe	Russia
C6	Brown eyes Black hair Black skin	No data Black hair Black skin	High: Africa Likely: Central/West	Burkina Faso
C7	Brown eyes Black hair Black skin	Brown eyes Black hair Black skin	ADMIXED (SWA-AFR) Likely: East Africa	Eritrea
C8	Brown eyes Black hair Dark skin	Brown eyes Black hair Dark skin	ADMIXED (SWA-AFR) Likely: East Africa	Ethiopia
C9	Brown eyes Black hair Intermediate skin	Brown eyes Black hair Intermediate skin	High: Asia High: East Asia	China
C10	Brown eyes Black hair Intermediate skin	Brown eyes Black hair Intermediate skin	High: Asia High: East Asia	Vietnam
C11	Blue eyes Blond to light blond hair Pale to intermediate skin	No data (body decayed)	High: Europe	Brazil
C12	Brown eyes Black hair Dark skin	No data (body decayed)	ADMIXED (SWA-EU-SA) Likely: Southwest Asia	Iraq
C13	Brown eyes Black hair Black skin	No data Black hair Black skin	High; Africa Likely: Central/West	Nigeria
C14	Brown eyes Brown to dark brown hair Dark skin to black skin	No data Dark greying hair No data	ADMIXED (EU-SA-OCE)	Afghanistan
C15	Brown eyes Black hair Intermediate to dark skin	No data Black hair Intermediate skin	ADMIXED (AME-SWA-AFR) Likely: South America	Mexico
C16	Brown eyes Dark brown to black hair Pale to intermediate skin	No data Dark greying hair Intermediate skin	ADMIXED (SWA-EU-SA) Likely: Southwest Asia	Iran
C17	Brown eyes Black hair Intermediate skin	No data Dark greying hair Intermediate skin	High: Asia High: East Asia	Japan

## Supplementary Materials

The following are available online at https://www.mdpi.com/2073-4425/11/12/1398/s1, Table S1: The list of autosomal SNPs used in the panel design, Table S2: The list of the Y-chromosomal SNPs used in the panel design, Table S3: Mean coverage obtained for autosomal markers through the sensitivity study, Table S4: Mean coverage obtained for Y-chromosomal markers through the sensitivity study, Table S5: Mean coverage obtained for autosomal markers for casework samples, Table S6: Mean coverage obtained for Y-chromosomal markers for casework samples, Figure S1: Heatmaps summarizing the performance of the panel based on the consensus genotypes/haplotypes obtained through the sensitivity study. Table S7: Detailed performance of the autosomal markers for calling genotypes.
