# Supplementary figures and images for: Evaluation of the Ion AmpliSeq™ PhenoTrivium Panel: MPS-Based Assay for Ancestry and Phenotype Predictions Challenged by Casework Samples
Source: Genes (Basel). 2020 Nov 25;11(12):1398. doi: 10.3390/genes11121398 (PMC7760956; doi:10.3390/genes11121398)

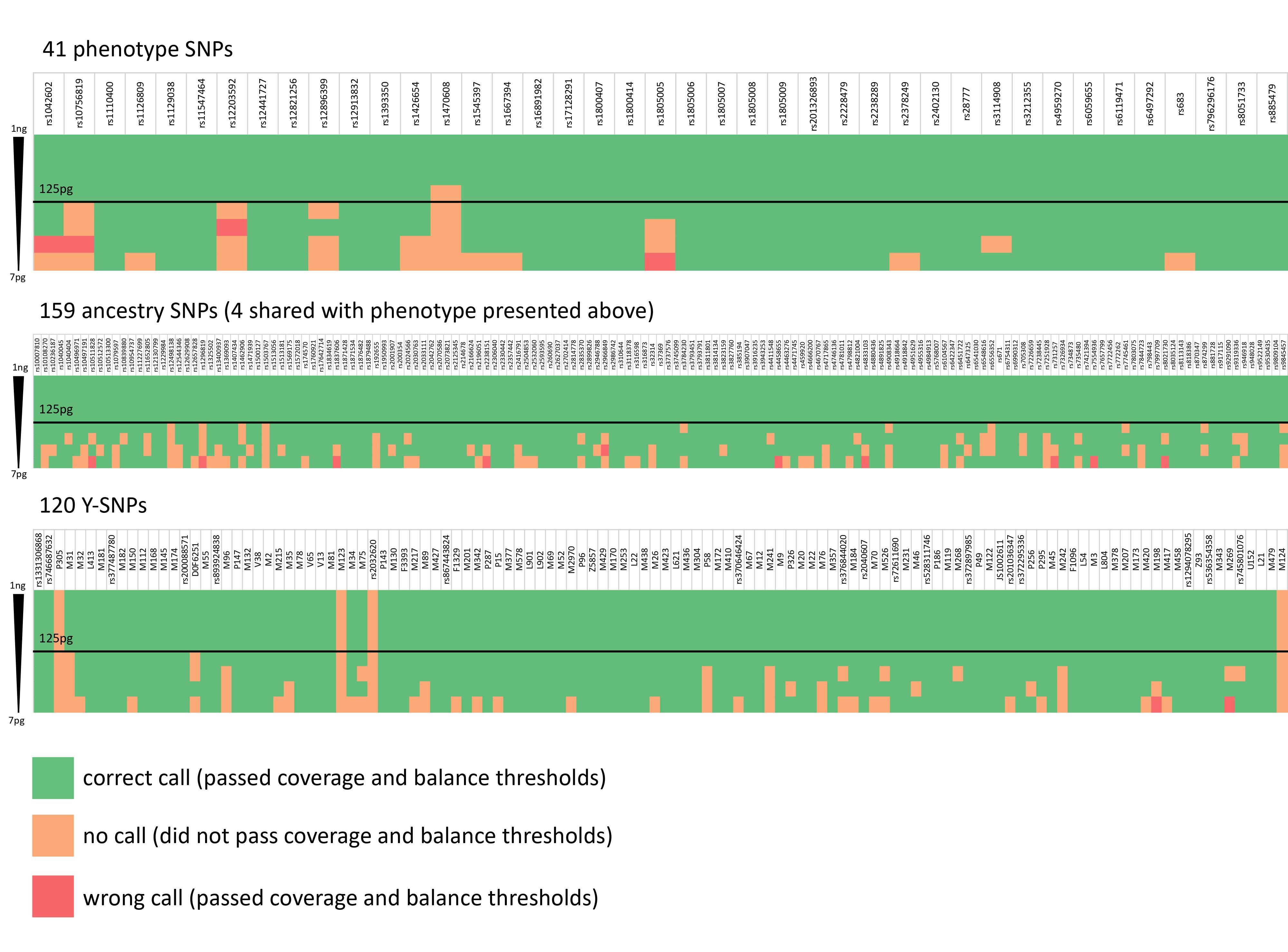

Supplement: Supplementary file 1 [file genes-11-01398-s001.zip › Supplementary Files/Figure S1.jpg]
