# Supplementary material for: Evaluation of the Ion AmpliSeq™ PhenoTrivium Panel: MPS-Based Assay for Ancestry and Phenotype Predictions Challenged by Casework Samples
Source: Genes (Basel). 2020 Nov 25;11(12):1398. doi: 10.3390/genes11121398 (PMC7760956; doi:10.3390/genes11121398)
Supplement: Supplementary file 1 [file genes-11-01398-s001.zip › Supplementary Files/Table S1-S2.doc]

Table S1. The list of autosomal SNPs used in the panel design (in yellow are marked the SNPs used by Converge for admixture analysis)

| Name | Chromosome | Position GRCh37 | Associated with |
| --- | --- | --- | --- |
| rs10007810 | 4 | 41554364 | ancestry |
| rs10108270 | 8 | 4190793 | ancestry |
| rs10236187 | 7 | 139447377 | ancestry |
| rs1040045 | 6 | 4747159 | ancestry |
| rs1040404 | 1 | 168159890 | ancestry |
| rs1042602 | 11 | 88911696 | phenotype |
| rs10496971 | 2 | 145769943 | ancestry |
| rs10497191 | 2 | 158667217 | ancestry |
| rs10511828 | 9 | 28628500 | ancestry |
| rs10512572 | 17 | 69512099 | ancestry |
| rs10513300 | 9 | 120130206 | ancestry |
| rs10756819 | 9 | 16858084 | phenotype |
| rs1079597 | 11 | 113296286 | ancestry |
| rs10839880 | 11 | 7850316 | ancestry |
| rs10954737 | 7 | 83533047 | ancestry |
| rs1110400 | 16 | 89986130 | phenotype |
| rs11227699 | 11 | 66898492 | ancestry |
| rs1126809 | 11 | 89017961 | phenotype |
| rs1129038 | 15 | 28356859 | phenotype |
| rs11547464 | 16 | 89986091 | phenotype |
| rs11652805 | 17 | 62987151 | ancestry |
| rs12130799 | 1 | 55663372 | ancestry |
| rs12203592 | 6 | 396321 | phenotype |
| rs1229984 | 4 | 100239319 | ancestry |
| rs12441727 | 15 | 28271775 | phenotype |
| rs12498138 | 3 | 121459589 | ancestry |
| rs12544346 | 8 | 86424616 | ancestry |
| rs12629908 | 3 | 120522716 | ancestry |
| rs12657828 | 5 | 79085726 | ancestry |
| rs12821256 | 12 | 89328335 | phenotype |
| rs12896399 | 14 | 92773663 | phenotype |
| rs12913832 | 15 | 28365618 | ancestry, phenotype |
| rs1296819 | 22 | 18076546 | ancestry |
| rs1325502 | 1 | 42360270 | ancestry |
| rs13400937 | 2 | 79864923 | ancestry |
| rs1369093 | 4 | 73245191 | ancestry |
| rs1393350 | 11 | 89011046 | phenotype |
| rs1407434 | 1 | 186149032 | ancestry |
| rs1426654 | 15 | 48426484 | ancestry, phenotype |
| rs1462906 | 8 | 31896592 | ancestry |
| rs1470608 | 15 | 28288121 | phenotype |
| rs1471939 | 8 | 28941305 | ancestry |
| rs1500127 | 5 | 165739982 | ancestry |
| rs1503767 | 12 | 118889488 | ancestry |
| rs1513056 | 12 | 17407792 | ancestry |
| rs1513181 | 3 | 188574996 | ancestry |
| rs1545397 | 15 | 28187772 | phenotype |
| rs1569175 | 2 | 201021954 | ancestry |
| rs1572018 | 13 | 41715282 | ancestry |
| rs1667394 | 15 | 28530182 | phenotype |
| rs16891982 | 5 | 33951693 | phenotype, ancestry |
| rs17128291 | 14 | 92882826 | phenotype |
| rs174570 | 11 | 61597212 | ancestry |
| rs1760921 | 14 | 20818131 | ancestry |
| rs17642714 | 17 | 48726132 | ancestry |
| rs1800407 | 15 | 28230318 | phenotype |
| rs1800414 | 15 | 28197037 | ancestry, phenotype |
| rs1805005 | 16 | 89985844 | phenotype |
| rs1805006 | 16 | 89985918 | phenotype |
| rs1805007 | 16 | 89986117 | phenotype |
| rs1805008 | 16 | 89986144 | phenotype |
| rs1805009 | 16 | 89986546 | phenotype |
| rs1834619 | 2 | 17901485 | ancestry |
| rs1837606 | 11 | 15838137 | ancestry |
| rs1871428 | 6 | 168665760 | ancestry |
| rs1871534 | 8 | 145639681 | ancestry |
| rs1876482 | 2 | 17362568 | ancestry |
| rs1879488 | 17 | 1401613 | ancestry |
| rs192655 | 6 | 90518278 | ancestry |
| rs1950993 | 14 | 58238687 | ancestry |
| rs2001907 | 8 | 140241181 | ancestry |
| rs200354 | 14 | 99375321 | ancestry |
| rs201326893 | 16 | 89986122 | phenotype |
| rs2024566 | 22 | 41697338 | ancestry |
| rs2030763 | 3 | 179964727 | ancestry |
| rs2033111 | 17 | 53788280 | ancestry |
| rs2042762 | 18 | 35277622 | ancestry |
| rs2070586 | 12 | 109277720 | ancestry |
| rs2073821 | 9 | 135933122 | ancestry |
| rs2125345 | 17 | 73782191 | ancestry |
| rs214678 | 12 | 47676950 | ancestry |
| rs2166624 | 13 | 42579985 | ancestry |
| rs2196051 | 8 | 122124302 | ancestry |
| rs2228479 | 16 | 89985940 | phenotype |
| rs2238151 | 12 | 112211833 | ancestry |
| rs2238289 | 15 | 28453215 | phenotype |
| rs2306040 | 9 | 93641199 | ancestry |
| rs2330442 | 7 | 42380071 | ancestry |
| rs2357442 | 14 | 52607967 | ancestry |
| rs2378249 | 20 | 33218090 | phenotype |
| rs2402130 | 14 | 92801203 | phenotype |
| rs2416791 | 12 | 11701488 | ancestry |
| rs2504853 | 6 | 12535111 | ancestry |
| rs2532060 | 19 | 55614923 | ancestry |
| rs2593595 | 17 | 41056245 | ancestry |
| rs260690 | 2 | 109579738 | ancestry |
| rs2627037 | 2 | 179606538 | ancestry |
| rs2702414 | 4 | 179399523 | ancestry |
| rs2814778 | 1 | 159174683 | ancestry |
| rs2835370 | 21 | 37885625 | ancestry |
| rs28777 | 5 | 33958959 | phenotype |
| rs2899826 | 15 | 74734500 | ancestry |
| rs2946788 | 11 | 24010530 | ancestry |
| rs2966849 | 16 | 85183682 | ancestry |
| rs2986742 | 1 | 6550376 | ancestry |
| rs310644 | 20 | 62159504 | ancestry |
| rs3114908 | 16 | 89383725 | phenotype |
| rs3118378 | 1 | 68849687 | ancestry |
| rs316598 | 5 | 2364626 | ancestry |
| rs316873 | 1 | 242342504 | ancestry |
| rs3212355 | 16 | 89984378 | phenotype |
| rs32314 | 7 | 32179124 | ancestry |
| rs37369 | 5 | 35037115 | ancestry |
| rs3737576 | 1 | 101709563 | ancestry |
| rs3745099 | 19 | 52901905 | ancestry |
| rs3784230 | 14 | 105679055 | ancestry |
| rs3793451 | 9 | 71659280 | ancestry |
| rs3793791 | 10 | 50511710 | ancestry |
| rs3811801 | 4 | 100244319 | ancestry |
| rs3814134 | 9 | 127267689 | ancestry |
| rs3823159 | 6 | 136482727 | ancestry |
| rs3827760 | 2 | 109513601 | ancestry |
| rs385194 | 4 | 85309078 | ancestry |
| rs3907047 | 20 | 54000914 | ancestry |
| rs3916235 | 18 | 67578931 | ancestry |
| rs3943253 | 8 | 13359500 | ancestry |
| rs4411548 | 17 | 40658533 | ancestry |
| rs4458655 | 6 | 163221792 | ancestry |
| rs4463276 | 6 | 145055331 | ancestry |
| rs4471745 | 17 | 53568884 | ancestry |
| rs459920 | 16 | 89730827 | ancestry |
| rs4666200 | 2 | 29538411 | ancestry |
| rs4670767 | 2 | 37941396 | ancestry |
| rs4717865 | 7 | 73454199 | ancestry |
| rs4746136 | 10 | 74971000 | ancestry |
| rs4781011 | 16 | 10975311 | ancestry |
| rs4798812 | 18 | 9420504 | ancestry |
| rs4821004 | 22 | 32366359 | ancestry |
| rs4833103 | 4 | 38815502 | ancestry |
| rs4880436 | 10 | 134500093 | ancestry |
| rs4891825 | 18 | 67867663 | ancestry |
| rs4908343 | 1 | 27931698 | ancestry |
| rs4918664 | 10 | 94911055 | ancestry |
| rs4918842 | 10 | 115306802 | ancestry |
| rs4951629 | 1 | 212786883 | ancestry |
| rs4955316 | 3 | 30415612 | ancestry |
| rs4959270 | 6 | 457748 | phenotype |
| rs4984913 | 16 | 740466 | ancestry |
| rs5768007 | 22 | 48207872 | ancestry |
| rs6059655 | 20 | 32665748 | phenotype |
| rs6104567 | 20 | 10195433 | ancestry |
| rs6119471 | 20 | 32785212 | phenotype |
| rs6422347 | 5 | 177863083 | ancestry |
| rs6451722 | 5 | 43711378 | ancestry |
| rs647325 | 1 | 18170886 | ancestry |
| rs6497292 | 15 | 28496195 | phenotype |
| rs6541030 | 1 | 12608178 | ancestry |
| rs6548616 | 3 | 79399575 | ancestry |
| rs6556352 | 5 | 155471714 | ancestry |
| rs671 | 12 | 112241766 | ancestry |
| rs6754311 | 2 | 136707982 | ancestry |
| rs683 | 9 | 12709305 | phenotype |
| rs6990312 | 8 | 110602317 | ancestry |
| rs705308 | 7 | 97695363 | ancestry |
| rs7226659 | 18 | 40488279 | ancestry |
| rs7238445 | 18 | 49781544 | ancestry |
| rs7251928 | 19 | 4077096 | ancestry |
| rs731257 | 7 | 12669251 | ancestry |
| rs7326934 | 13 | 49070512 | ancestry |
| rs734873 | 3 | 147750355 | ancestry |
| rs735480 | 15 | 45152371 | ancestry |
| rs7421394 | 2 | 14756349 | ancestry |
| rs7554936 | 1 | 151122489 | ancestry |
| rs7657799 | 4 | 105375423 | ancestry |
| rs7722456 | 5 | 170202984 | ancestry |
| rs772262 | 12 | 56163734 | ancestry |
| rs7745461 | 6 | 21911616 | ancestry |
| rs7803075 | 7 | 130742066 | ancestry |
| rs7844723 | 8 | 122908503 | ancestry |
| rs796296176 | 16 | 89985750 | phenotype |
| rs798443 | 2 | 7968275 | ancestry |
| rs7997709 | 13 | 34847737 | ancestry |
| rs8021730 | 14 | 67886781 | ancestry |
| rs8035124 | 15 | 92105708 | ancestry |
| rs8051733 | 16 | 90024206 | phenotype |
| rs8113143 | 19 | 33652247 | ancestry |
| rs818386 | 16 | 65406708 | ancestry |
| rs870347 | 5 | 6845035 | ancestry |
| rs874299 | 18 | 75056284 | ancestry |
| rs881728 | 18 | 59333108 | ancestry |
| rs885479 | 16 | 89986154 | phenotype |
| rs917115 | 7 | 28139111 | ancestry |
| rs9291090 | 4 | 5390637 | ancestry |
| rs9319336 | 13 | 27624356 | ancestry |
| rs946918 | 14 | 83472868 | ancestry |
| rs948028 | 11 | 120644447 | ancestry |
| rs9522149 | 13 | 111827167 | ancestry |
| rs9530435 | 13 | 75993887 | ancestry |
| rs9809104 | 3 | 39146429 | ancestry |
| rs9845457 | 3 | 135914476 | ancestry |

Table S2. The list of Y-chromosomal SNPs used in the panel design

| SNP ID | Position | Ancestral allele | Derived allele | Haplogroup |
| --- | --- | --- | --- | --- |
| AF6 | 6877771 | A | G | A00 |
| CTS2809 | 14497059 | C | A | A0 |
| P305 | 2710154 | A | G | A1 |
| M31 | 21739754 | G | C | A1a |
| M32 | 21740436 | T | C | A1b1b |
| L413 | 6932831 | G | A | BT |
| M181 | 14851554 | T | C | B |
| M146 | 21779251 | T | G | B1a |
| M182 | 14869076 | C | T | B2 |
| M150 | 21869519 | C | T | B2a |
| M112 | 21767959 | C | T | B2b |
| M168 | 14813991 | C | T | CT |
| M145 | 21717208 | C | T | DE |
| M174 | 14954280 | T | C | D1 |
| CTS11577 | 23165423 | C | A | D1a |
| F6251 | 7681275 | C | T | D1a1 |
| M55 | 21872738 | T | C | D1a2 |
| L1378 | 2828140 | C | T | D1b |
| M96 | 21778998 | C | G | E |
| P147 | 21083420 | T | A | E1 |
| M132 | 21896261 | G | T | E1a |
| V38 | 6818291 | C | T | E1b1a |
| M2 | 14096577 | A | G | E1b1a1 |
| M215 | 15467824 | A | G | E1b1b |
| M35 | 21741703 | G | C | E1b1b1 |
| M78 | 21893303 | C | T | E1b1b1a1 |
| V65 | 17287868 | C | A | E1b1b1a1a2 |
| V13 | 6842263 | G | A | E1b1b1a1b1a |
| M81 | 21892572 | C | T | E1b1b1b1a |
| M123 | 21764586 | C | T | E1b1b1b2a1 |
| M34 | 21740716 | G | T | E1b1b1b2a1a |
| M75 | 21890177 | G | A | E2 |
| M54 | 21869264 | G | A | E2b1 |
| P143 | 14197867 | A | A | CF |
| M130 | 2734854 | C | T | C |
| F3393 | 23023974 | C | A | C1 |
| M217 | 15437333 | A | C | C2 |
| M89 | 21917313 | C | T | F |
| M427 | 19092597 | G | A | F2 |
| M481 | 14496387 | C | T | F3 |
| F1329 | 8589031 | C | T | GHIJK |
| M201 | 15027529 | G | T | G |
| M342 | 23243942 | C | T | G1 |
| P287 | 22072097 | G | T | G2 |
| P15 | 23244026 | C | T | G2a |
| M377 | 15027433 | A | G | G2b1 |
| M578 | 7202703 | C | T | HIJK |
| L901 | 17844304 | C | T | H |
| L902 | 23009978 | C | T | H1 |
| M69 | 21894058 | T | C | H1a |
| M52 | 21753199 | A | C | H1a1 |
| M2970 | 18870223 | T | G | H1a1a |
| P96 | 14869743 | C | A | H2 |
| Z5857 | 2759285 | C | G | H3 |
| M429 | 14031334 | T | A | IJ |
| M170 | 14847792 | A | C | I |
| M253 | 15022707 | C | T | I1 |
| L22 | 8576009 | A | C | I1a1b1 |
| M438 | 16638804 | A | G | I2 |
| M26 | 21865821 | G | A | I2a1a1a |
| M423 | 19096091 | G | A | I2a1a2 |
| L621 | 18760081 | G | A | I2a1a2b |
| M436 | 18747493 | G | C | I2a1b |
| M304 | 22749853 | A | C | J |
| P58 | 14486667 | T | C | J1a2a1a2 |
| M172 | 14969634 | T | G | J2 |
| M410 | 2751678 | A | G | J2a |
| M47 | 21751330 | G | A | J2a1a1a2a2b |
| M67 | 21878809 | A | T | J2a1a1a2b2 |
| M12 | 7583480 | G | T | J2b |
| M241 | 15018459 | G | A | J2b2a |
| M9 | 21730257 | C | G | K |
| P326 | 8467290 | T | C | LT (K1) |
| M20 | 21733454 | A | G | L (K1a) |
| M22 | 14790163 | A | G | L1 |
| M76 | 21757316 | T | G | L1a1 |
| M357 | 2888252 | C | A | L1a2 |
| L655 | 4874269 | C | T | L1b |
| M184 | 14898163 | G | A | T (K1b) |
| rs2040607 | 14529630 | G | A | T1 |
| M70 | 21893881 | A | C | T1a |
| M526 | 23550924 | A | C | K2 |
| F549 | 19513070 | C | T | NO (K2a) |
| M231 | 15469724 | G | A | N (K2a1a) |
| M46 | 14922583 | T | C | N1a1 |
| F2930 | 19080602 | G | A | N1b |
| P186 | 7568568 | C | A | O (K2a1b) |
| M119 | 21762685 | T | G | O1a |
| M268 | 22739301 | A | G | O1b |
| F2320 | 16903082 | G | C | O1b1 |
| P49 | 14496407 | A | T | O1b2 |
| M122 | 21764674 | A | G | O2 |
| JST002611 | 7546726 | G | A | O2a1b |
| rs201036347 | 7809769 | A | G | O2a2b1 |
| B254 | 14102580 | C | A | S (K2b1a) |
| P256 | 8685231 | G | A | M (K2b1b) |
| P295 | 7963031 | T | G | P (K2b2) |
| M45 | 21867787 | G | A | P1 (K2b2a) |
| M242 | 15018582 | C | T | Q (K2b2a1) |
| F1096 | 8087573 | A | T | Q1a |
| L54 | 23292782 | G | A | Q1b1a |
| M3 | 19096363 | G | A | Q1b1a1a |
| L804 | 14028228 | T | A | Q1b1a1b |
| M378 | 15027507 | A | G | Q2a1 |
| M207 | 15581983 | A | G | R (K2b2a2) |
| M173 | 15026424 | A | C | R1 |
| M420 | 23473201 | T | A | R1a |
| M198 | 15030752 | C | T | R1a1a |
| M417 | 8533735 | G | A | R1a1a1 |
| M458 | 24366464 | A | G | R1a1a1b1a1a |
| Z280 | 6229881 | C | T | R1a1a1b1a2 |
| Z93 | 7552356 | G | A | R1a1a1b2 |
| F1345 | 8605676 | C | A | R1a1a1b2a2b1 |
| M343 | 2887824 | C | A | R1b |
| M269 | 22739367 | T | C | R1b1a1b |
| U152 | 19315298 | C | T | R1b1a1b1a1a2b |
| L21 | 15333149 | C | G | R1b1a1b1a1a2c1 |
| M479 | 15654428 | C | T | R2 |
| M124 | 20834667 | G | A | R2a |
